# Phenotypic and Genomic Characterization of *Streptomyces* sp. HR58 Reveals Its Potential Against Fungal Phytopathogens

**DOI:** 10.3390/microorganisms14102344

**Published:** 2026-10-14

**Authors:** Carla Calvo-Peña, Marina Ruiz-Muñoz, Seyedehtannaz Ghoreshizadeh, Samantha Crespo, Juan José R. Coque, Rebeca Cobos

**Affiliations:** Instituto de Investigación de la Viña y el Vino, Escuela de Ingeniería Agraria, Universidad de León, 24009 León, Spain; ccalp@unileon.es (C.C.-P.); mruim@unileon.es (M.R.-M.); sghore00@estudiantes.unileon.es (S.G.); scresg01@estudiantes.unileon.es (S.C.)

**Keywords:** antifungal activity, biological control, biosynthetic gene clusters, phytopathogenic fungi, secondary metabolites, silver nanoparticles

## Abstract

*Streptomyces* sp. HR58, isolated from the hop rhizosphere, exhibits antifungal activity against a variety of fungal phytopathogens affecting many crops. HR58 was characterized using phenotypic and genomic approaches. Growth tolerance and enzymatic activities were assessed, and whole-genome sequencing enabled the prediction of biosynthetic gene clusters. The strain HR58 displayed morphological and biochemical traits characteristic of the genus *Streptomyces*, growing from pH 5 to pH 12 and salt tolerance up to 5% NaCl. Genome analysis revealed a size of 8,270,372 bp with a GC content of 71.76% and the presence of gene clusters associated with antifungal activity. Although ANI analysis showed 96.6% identity with *Streptomyces anulatus* ATCC 11523, the dDDH value of 66.5% fell below the species delineation threshold of 70%, indicating that HR58 cannot be confidently assigned to any currently described *Streptomyces* species. A biosynthetic gene cluster encoding the antifungal compound cycloheximide was identified in its genome, and cycloheximide production was assessed in liquid cultures. Silver nanoparticle (AgNP) biosynthesis by strain HR58 was also confirmed, and its antimicrobial activity was evaluated. The ability of strain HR58 to biosynthesize antifungal nanoparticles further enhances its potential applications in sustainable agriculture, offering a promising alternative for the development of eco-friendly plant disease control strategies.

## 1. Introduction

Fungal phytopathogens are among the major constraints to agricultural productivity worldwide, causing severe yield losses and threatening the sustainability of cropping systems. Due to climate change, their incidence is expected to continue increasing in the coming decades [1]. Conventional chemical control is increasingly limited by regulatory restrictions, environmental concerns, and the rapid emergence of fungicide resistance [2], highlighting the need for novel, effective and sustainable disease management strategies.

Microbial antagonists (biocontrol agents) have emerged as promising alternatives within integrated pest management, providing effective and environmentally sustainable control of plant pests and pathogens while reducing dependence on chemical pesticides [3]. Among them, the genus *Streptomyces* stands out due to its exceptional biosynthetic versatility. *Streptomyces* spp. are well known for their ability to produce a wide variety of bioactive compounds with antifungal, antibacterial and plant growth–promoting properties [4,5]. Many species also behave as beneficial endophytes and rhizosphere microorganisms, enhancing plant health through antibiosis, nutrient mobilization, enzymatic degradation of fungal cell walls and modulation of the rhizosphere microbiome [6].

Advances in whole-genome sequencing and comparative genomics have substantially improved the taxonomic resolution and functional characterization of *Streptomyces* species, enabling the identification of genome features, phylogenetic relationships and biosynthetic gene clusters (BGCs) involved in secondary metabolite production [7]. In addition to their well-known ability to synthesize bioactive compounds, some *Streptomyces* species have recently been explored as biological factories for the green synthesis of metallic nanoparticles, linking microbial biotechnology with nanotechnology for agricultural and biomedical applications.

Nanoparticles are at the leading edge of the rapidly developing field of nanotechnology, and they are currently produced by chemical, physical and biological methods. Different elements, such as magnesium, zinc, copper, gold, platinum and silver, are used for nanoparticle synthesis. However, unique properties of silver nanoparticles (AgNPs) at the nanoscale, such as high surface plasmon resonance (SPR), high stability, good conductivity, photoelectrochemical activity and high activity against pathogenic microbes, provide more biological applications [8]. Several reports are available for biosynthesis of AgNPs using different *Streptomyces* species, such as *S. atrovirens* [9], *S. glaucus* [10], *S. griseorubens* [11], *S. griseoplanus* [12], *S. hygroscopicus* [13], *S. catenulae* [14], *S. capillispiralis*, *S. zaomyceticus* and *S. pseudogriseolus* [8].

In this study, we performed an extensive characterization of *Streptomyces* sp. HR58, a promising biocontrol agent, including its phenotypic traits, phylogenomic placement and biosynthetic potential. We also evaluated its antifungal activity against a broad panel of agriculturally relevant fungal pathogens. Furthermore, we investigated the production of antifungals and the strain’s ability to synthesize silver nanoparticles, as well as the antifungal properties of the resulting AgNPs. Overall, this work provides an integrated assessment of the biocontrol potential of HR58 and highlights its value as a source of bioactive metabolites with applications in sustainable plant disease management.

## 2. Materials and Methods

### 2.1. Strains and Growth Conditions

*Streptomyces* sp. HR58 was isolated from the rhizosphere of hop plants (Nugget cultivar) cultivated under conventional agronomic practices in a field located in León, Spain [15]. Rhizosphere soil samples were collected from five individual plants by carefully excavating the surrounding root systems. Soil in direct contact with the roots was obtained using a sterile spatula, transferred into sterile 50 mL Falcon tubes, and stored in an icebox prior to preservation at 4 °C until further processing. Culturable streptomycetes were isolated on starch casein agar (SCA) [16] and International Streptomyces Project 2 (ISP2) agar medium [17]. The strain was routinely cultivated on MEY (Maltose Yeast Extract) medium at 30 °C and maintained at 4 °C [18].

*Alternaria alternata*, *Cadophora luteo-olivacea*, *Dactylonectria macrodydima*, *Diplodia seriata*, *Fusarium fujikuroi*, *F. oxysporum*, *Phaeomoniella chlamydospora*, *Pleurostoma richardsiae*, *Rhizoctonia solani* and *Thelonectria olida* were isolated from diseased grapevines. *Verticillium dahliae* D0913 and *V. dahliae* ND1113 were kindly provided by Dr. Leire Molinero [19] and *V. dahliae* V937I was kindly provided by Dr. Jesús Mercado [20], having been originally isolated from olive trees. Fungal isolates were routinely cultivated on PDA (Potato Dextrose agar) medium at 25 °C and maintained at 4 °C.

Pure cultures of bacteria (*Bacillus subtilis*, *Enterococcus faecalis*, *Escherichia coli* and *Salmonella typhimurium*) were grown in Nutrient Broth medium at 37 °C on a rotary shaker at 200 rpm. Yeasts (*Candida albicans* and *Saccharomyces cerevisiae*) were grown in YEPD medium at 28 °C on a rotary shaker at 180 rpm.

For long-term storage, spore or cell suspensions were preserved in 40% glycerol at −20 °C.

### 2.2. Phenotypic Characterization of Streptomyces sp. HR58

The cultural characteristics of strain HR58 were evaluated following the phenotypic characterization procedure described by Calvo-Peña et al. [21] The strain was grown on a range of solid media, including ISP 1 (DSMZ 1764), ISP 2 (DSMZ 987), ISP 3 (DSMZ 609), ISP 4 (DSMZ 547), ISP 5 (DSMZ 993), ISP 6 (DSMZ 1269), ISP 7 (DSMZ 1619) [17], nutrient agar (DSMZ 1) (Scharlab S.L., Sentmenat, Spain), Bennett’s agar (DSMZ 548; HiMedia Laboratories GmbH, Modautal, Germany), trypticase soy agar (TSA; DSMZ 535; Scharlab S.L., Sentmenat, Spain), R5 agar [18] and GYM medium (DSMZ 65). Cultures were incubated at 28 °C for 7 days before colony morphology was examined. Growth was assessed at temperatures ranging from 4 to 45 °C and at pH values between 5.0 and 12.0 using GYM medium (glucose 4 g/L; yeast extract, 4 g/L; malt extract, 10 g/L; CaCO_3_, 2 g/L; agar, 20 g/L; pH 7.2). Salt tolerance was determined on ISP2 medium supplemented with NaCl concentrations ranging from 2.5% to 7.5% (*w*/*v*). Colony pigmentation, including the color of aerial and substrate mycelia and the production of diffusible pigments, was recorded using a RAL colour chart. All tests were conducted in triplicate.

The biochemical and enzymatic properties of strain HR58 were characterized using API-ZYM and API 20NE test strips (bioMerieux, Lyon, France). For API 20NE, inoculation of the cupules was performed at 28 °C with a bacterial suspension standardized to a turbidity of 5 on the McFarland scale, and results were recorded at 24 and 48 h. API-ZYM assays were conducted at 28 °C instead of the standard 37 °C, with an incubation period of 24 h. Both tests were conducted in duplicate.

### 2.3. Genome Features and Phylogenomic Analysis

Whole-genome sequencing of strain HR58 was performed by Macrogen Inc. (Seoul, Republic of Korea) using PacBio Revio technology (Menlo Park, CA, USA) to generate long reads. Raw sequencing reads were assembled *de novo* using Canu v2.2 [22] with default parameters optimized for long-read data. Assembly quality metrics were assessed using QUAST v5.2.0 [23], and genome completeness was further evaluated with BUSCO v5.4.7 [24] using the actinobacteria_odb10 dataset. Functional genome annotation was performed with the RASTtk (RAST, Rapid Annotation using Subsystem Technology, http://rast.nmpdr.org; accesed on 18 January 2026) server [25].

The 16S rRNA gene sequence was extracted from the assembled genome and compared against type strain sequences in the NCBI RefSeq database using BLASTN v2.15.0+ [26]. Whole-genome relatedness was assessed through Average Nucleotide Identity (ANI) calculations using FastANI v1.34 [27] and digital DNA–DNA hybridization (dDDH) using the Type (Strain) Genome Server (TYGS) [28]. Thresholds of 95% ANI [27] and 70% dDDH were applied for species delineation [29]. Phylogenomic analysis was conducted within the TYGS framework using the Genome BLAST Distance Phylogeny (GBDP) method [30], with branch support estimated from 100 pseudo-bootstrap replicates.

### 2.4. Analysis of Bioactive Compound Biosynthetic Gene Clusters

Biosynthetic gene clusters (BGCs) were identified using antiSMASH version 7.1.0 [31], with all optional annotation modules enabled, including ClusterBlast, KnownClusterBlast, SubClusterBlast and ActiveSiteFinder. Predicted clusters were examined to determine cluster boundaries and their similarity to previously characterized biosynthetic pathways.

For comparative purposes, the genome of *S. anulatus* ATCC 11523 was analysed using the same antiSMASH version, detection strictness and optional modules applied to HR58. The comparison considered the total number of predicted regions, BGC classes, known-cluster assignments and the presence of canonical biosynthetic genes.

Genes encoding enzymes potentially involved in fungal cell wall degradation were identified by screening the RAST annotation using keyword- and EC number-based searches. Terms included “chitinase”, “chitin-binding protein”, “β-glucosidase”, “glucanase”, “cellulase” and “protease”. All predicted proteins associated with chitin and N-acetylglucosamine utilization subsystems were extracted and manually curated. Putative enzymes lacking formal EC annotations were retained when their conserved domain architecture (e.g., GH18 endochitinase-like signatures) supported their functional assignment. Signal peptide prediction was performed using SignalP version 6.0 with the “other organisms” model to identify proteins potentially secreted via the Sec or Tat pathways [32].

To explore additional functional traits potentially associated with rhizosphere competence and plant growth promotion, the genome annotation was screened for genes involved in siderophore biosynthesis and transport, phosphorus acquisition, ACC deamination, oxidative-stress tolerance and extracellular hydrolytic activity. The predicted proteome was also searched for oxidoreductases, flavin-dependent enzymes, thioredoxin-related proteins and other redox-active proteins that could potentially participate in the extracellular reduction of Ag(I).

### 2.5. Antifungal Activity of Streptomyces sp. HR58

In order to quantify the antifungal activity of *Streptomyces* sp. HR58 against fungal phytopathogens, the bacterial isolate was individually tested against each fungal pathogen on 9-cm-diameter MEY agar plates following Calvo-Peña et al. [33]. Briefly, a circle was drawn 1 cm from the edge of each plate, and 10^8^ CFU were inoculated within the marked area using a sterile swab. The plates were incubated at 30 °C for 48 h prior to inoculation with the fungal pathogen. Subsequently, a 6-mm-diameter agar plug containing the fungal pathogen was excised from a 10-day-old culture grown on PDA and placed at the centre of the plate. Plates were incubated at 25 °C for up to 10 days. The inhibition index (I index) was calculated as follows: I index (%) = [(Rc − R)/Rc] × 100, where R is the radius of the fungal colony in the presence of the bacterial isolate, and Rc is the maximum radius of the fungal colony in the control treatment. The well-characterized antifungal strain *S. lacaronensis* OE57 was included as a positive control and tested under the same experimental conditions. All tests were performed in triplicate for each fungal pathogen.

### 2.6. Purification and Identification of Cycloheximide Production

To assess cycloheximide production by the strain *Streptomyces* sp. HR58, an ethyl acetate extract was obtained from 100 mL of liquid culture (10^7^ CFU/mL) following the procedure described by Das et al. [34], and the crude extract was dissolved in 250 µL of 80% methanol.

Antifungal activity was evaluated using an in vitro plate assay against *V. dahliae* V937I. The assay was performed on PDA agar plates containing 0.8% agar (*w*/*v*), as described by Calvo-Peña et al. [33]. Briefly, 5.0 × 10^4^ fungal spores/mL were embedded in the agar medium before plating. Wells 6 mm in diameter were made in the agar plates, and each well was inoculated with 60 µL of crude extract. Plates were incubated at 25 °C for 4 days, and the diameters of the inhibition halos were measured. A total of 600 µg of commercial cycloheximide was added to one well as a control.

The remaining HR58 culture extract was filtered through Corning^®^ Costar^®^ Spin-X^®^ centrifuge tube filters (0.45 μm pore size; Merck KGaA, Darmstadt, Germany) and stored at −20 °C until further analysis. High-performance liquid chromatography (HPLC) analysis was performed using an Agilent 1200 Series Gradient HPLC System (Agilent Technologies, Santa Clara, CA, USA), following the chromatographic method described by Awla et al. [35]. The system was equipped with a quaternary pump delivery system (G1311A), a preparative autosampler (G1329A), a diode array multi-wavelength detector (G7115A) and an analytical fraction collector (G1364F) with an Autosampler Thermostat (G1330B). A 10 μL sample was injected and resolved using an analytical LiChrospher RP-18 column (40 × 250 mm; 5 μm) (Teknokroma, San Cugat del Vallés, Spain), with UV detection at 220 nm and a flow rate of 0.8 mL min^−1^. The mobile phase consisted of 0.1% formic acid in water (A) and methanol (B), with a gradient of 100–50–10–0–100% A at 0, 3, 12, 21 and 24 min, respectively, followed by re-equilibration at 50% A until 32 min.

### 2.7. Microbial Synthesis and Characterization of AgNPs

Nanoparticles were synthesized according to Sadhasivam et al. [13] and Vijayabharathi et al. [12]. Briefly, *Streptomyces* sp. HR58 was cultured in ISP2 broth at 28 °C with shaking at 200 rpm for 72 h. The culture (2 × 10^7^ CFU/mL) was subsequently centrifuged at 10,000 rpm for 15 min to remove the biomass, and the resulting supernatant was passed through a 0.2 µm membrane filter to obtain a sterile cell-free filtrate. For AgNP biosynthesis, the cell-free filtrate was mixed with a 1 mM AgNO_3_ solution (Thermo Scientific, Waltham, MA, USA) to obtain a final filtrate concentration of 10% (*v*/*v*). The reaction mixture was incubated at 28 °C and 200 rpm for 72 h in the absence of light.

The progress of nanoparticle formation was monitored every 24 h by UV–visible spectrophotometry (UV-1600PC, VWR, Leuven, Belgium). Absorption spectra were recorded between 300 and 800 nm using aliquots collected throughout the 72-h incubation period. Solutions containing only 1 mM AgNO_3_ or 10% (*v*/*v*) cell-free filtrate served as negative controls. Upon completion of the reaction, the synthesized AgNPs were recovered by centrifugation, thoroughly washed with nanopure water to remove unreacted components, freeze-dried, and stored for subsequent characterization and biological assays.

TEM specimens were prepared by placing a drop of the AgNPs suspension onto a Formvar-coated copper grid and allowing it to air-dry at room temperature. The size and morphology of the biosynthesized AgNPs were examined using a transmission electron microscope (TEM 1010, JEOL, Akishima, Japan) operated at an accelerating voltage of 90 kV [36].

### 2.8. Antibiotic Activity of AgNPs

The antimicrobial activity of the microbiologically synthesized AgNPs against pathogenic organisms such as Gram-positive bacteria (*Bacillus subtilis* and *Enterococcus faecalis*), Gram-negative bacteria (*Escherichia coli* and *Salmonella typhimurium*), yeasts (*Candida albicans* and *Saccharomyces cerevisiae*) and fungal phytopathogens (*Fusarium oxysporum*, *Alternaria alternata* and *Verticillium dahliae*) was measured using the well-diffusion method. Agar plates were prepared by adding 1 mL of an overnight culture of each microorganism to the medium, just before pouring it into the plates. Wells were made in the agar, and a 20-µL aliquot of the biosynthesized AgNPs solution was added to each well. After incubation, the diameter of the inhibition zone was measured. All tests were performed in triplicate.

### 2.9. Statistical Analysis

All statistical analyses were performed using R version 4.5.1 [37]. Radial-growth measurements were analysed separately for each fungal isolate. Two pre-specified comparisons were conducted: HR58 versus the fungus-only control and *S. lacaronensis* OE57 versus the fungus-only control. Two-sided Welch’s *t*-tests were used because homogeneity of variances could not be assumed. The resulting *p*-values were adjusted jointly across the 26 planned comparisons using the Holm method. Adjusted *p*-values < 0.05 were considered statistically significant.

For the AgNP dose–response assay, differences among treatment conditions were initially assessed using the Kruskal–Wallis test due to the small sample size, the presence of zero values, and the non-normal distribution of inhibition-zone measurements. Dunn’s tests were subsequently performed to compare each of the six AgNP doses with the water control and with 1 mM AgNO_3_. The Holm correction was applied separately to each family of six comparisons. Data are presented as mean ± standard deviation from three independent replicates.

## 3. Results

### 3.1. Phenotypic Characterization of Streptomyces sp. HR58

Morphological studies revealed that *Streptomyces* sp. HR58 grows well on different agar media, showing sporulation on most of the tested ISP media (Figure 1). As summarized in the Supplementary Material (Table S1), the strain exhibited a wide growth temperature range, growing well between 4 °C and 30 °C, but showing markedly reduced growth at 37 °C. It was able to grow at all pH values tested, from pH 5 to pH 12, indicating a broad tolerance to pH variation. Regarding salt tolerance, HR58 tolerated NaCl concentrations of 2.5% and 5%, whereas no growth was observed at a concentration of 7.5% NaCl. According to the RAL code, the strain exhibits a distinctive chromatic profile, with hues intermediate between papyrus white and green-beige, which contributes to its easy recognition among related taxa.

The biochemical profile obtained with the API 20NE and API-ZYM systems is detailed in the Supplementary Material (Table S2). In the API 20NE test, strong activity was observed for urease, esculin hydrolysis, gelatin hydrolysis and the assimilation of glucose, N-acetyl-glucosamine, potassium gluconate and malic acid. According to the API-ZYM test, *Streptomyces* sp. HR58 showed high activity for alkaline phosphatase, lipase (C14), leucine arylamidase, trypsin, α-chymotrypsin and α-glucosidase.

### 3.2. Genome Features and Phylogenomic Analysis

PacBio Revio long-read sequencing followed by Canu assembly generated a single contig of 8,270,372 bp with a GC content of 71.76%. QUAST analysis confirmed the structural integrity of the assembly. Genome annotation using RAST identified 7299 predicted protein-coding sequences, 84 RNA genes and 306 functional subsystems. BUSCO analysis indicated a high level of completeness for both the assembly and the predicted gene set, reaching 98.25% (Table 1).

The 16S rRNA gene sequence of strain HR58 showed 100% identity to members of the *Streptomyces anulatus* group, including *S. chrysomallus* and *S. citreofluorescens*. Average Nucleotide Identity (ANI) analysis revealed 96.6% nucleotide identity with *S. anulatus*, whereas digital DNA–DNA hybridization (dDDH) similarity between HR58 and *S. anulatus* ATCC 11523 (formerly *S. chrysomallus*) was 66.5%, with a confidence interval ranging from 63.5 to 69.3%, just below the 70% threshold used to distinguish between closely related species.

Genome-based phylogenomic analysis inferred from whole-genome sequences supported the affiliation of HR58 with the *Streptomyces anulatus* clade (Figure 2). In the GBDP-based phylogram, HR58 formed a distinct branch relative to the type strain and other closely related taxa, supported by high pseudo-bootstrap values (with an average branch support of 85.5%).

### 3.3. Analysis of Bioactive Compound Biosynthetic Gene Clusters

AntiSMASH v7.1.0 predicted a total of 35 biosynthetic gene clusters (BGCs) in the genome of *Streptomyces* sp. HR58, including NRPS, PKS, hybrid NRPS-PKS systems, RiPPs, terpenes, siderophores and other specialized metabolite pathways (Table 2; Supplementary Material Table S3).

Ten BGCs exhibited 100% similarity to previously characterized clusters, including those associated with geosmin, griseobactin, naringenin, ectoine, desferrioxamine B, melanin, AmfS, 2-methylisoborneol and SGR polycyclic tetramate macrolactams. Four additional BGCs exhibited high similarity (75–99%) to characterized pathways, including clusters related to skyllamycin D/E and warkmycin CS1/CS2. Among them, only naringenin (Region 4) and SGR PTM (Region 28) have been described as antifungal compounds [38,39].

A trans-AT polyketide synthase (PKS) region showing 94% similarity to the cycloheximide biosynthetic gene cluster was identified (Region 34, Table S3). The cluster contained the core biosynthetic genes typically associated with cycloheximide biosynthesis in *Streptomyces*, with minor differences in gene content and organization.

Three BGCs displayed moderate similarity (30–70%) to known pathways, whereas fourteen showed low similarity (1–29%). Four predicted BGCs showed no detectable similarity to characterized clusters and were classified as cryptic (Table 2).

Under the same analysis conditions, antiSMASH predicted 36 BGCs in *S. anulatus* ATCC 11523, compared with 35 in HR58. The two strains shared the main BGC classes and several known-cluster assignments. The most notable difference was an actinomycin D-related region in ATCC 11523 containing the biosynthetic genes *acmI*, *acmC*, *acmB*, *acmA*, *acmD* and *acmL* [40]. This gene set was not detected in HR58. Conserved genes flanking the ATCC 11523 actinomycin region were identified in HR58, but the intervening interval contained a distinct NRPS/PKS-associated region lacking the canonical *acm* genes.

The genome of *Streptomyces* sp. HR58 encodes enzymes predicted to target fungal cell wall components (Table 3). The chitinolytic repertoire includes eight chitinases (six annotated as EC 3.2.1.14 and two putative endochitinases), four chitin-binding proteins and two β-N-acetylglucosaminidases (EC 3.2.1.52). Four endo-1,4-β-glucanases were also identified. SignalP-6.0 predicted Sec/SPI signal peptides consistent with extracellular localization for seven of the eight chitinases.

In addition, HR58 encodes 62 predicted proteases, including 38 genes exceeding 1000 bp in length. SignalP analysis predicted secretion signals for 23 proteases via the Sec or Tat pathways. Genes encoding LysM-domain proteins, AA10/AA11 lytic polysaccharide monooxygenases, or canonical chitin-catabolic regulators (*dasR*, *chiR* and *nagR*) were not detected.

The functional annotation also revealed several traits potentially associated with rhizosphere competence and plant growth promotion. These included two putative ACC deaminases, several PhoD-type alkaline phosphatases, a broad-specificity PhoE phosphatase and multiple organic-phosphate transport components. Genes associated with siderophore biosynthesis, ferric-siderophore transport and intracellular iron release were also detected. Consistently, antiSMASH identified siderophore-related BGCs, including a desferrioxamine B-like region showing 80% similarity to the characterized reference cluster. These genomic features suggest potential capacities for phosphorus acquisition, modulation of plant stress responses and competition for iron (Table S6), although these activities were not experimentally assessed.

In addition, HR58 encoded several redox-related protein families, including NAD(P)H-dependent flavin oxidoreductases, ferric-siderophore reductases, nitroreductase-family proteins and thioredoxin-related proteins (Table S6). These functions provide plausible candidates for metabolic processes contributing to the reducing capacity of the cell-free filtrate. However, no specific genetic determinant of Ag(I) reduction could be identified, and their involvement in AgNPs formation remains hypothetical.

### 3.4. Antifungal Activity of Streptomyces sp. HR58

Antifungal activity was assessed using dual culture assays against eleven fungal species, represented by thirteen phytopathogen isolates affecting a wide range of crops: *A. alternata*, *C. luteo-olivacea*, *D. macrodydima*, *D. seriata*, *F. fujikuroi*, *F. oxysporum*, *P. chlamydospora*, *P. richardsiae*, *R. solani*, *T. olida;* and *V. dahlia* (Figure 3, Table S4). The Inhibition index (I-index) ranged from 25.7% against *Thelonectria olida* to 80.9% against *Verticillium dahliae* ND1113 (Figure 3). After Holm correction for multiple comparisons, radial growth was significantly lower in the presence of HR58 than in the corresponding fungus-only control for all isolates tested (adjusted *p* < 0.05; Table S4). *S. lacaronensis* OE57 also significantly reduced the radial growth of all fungal isolates relative to their respective controls (adjusted *p* < 0.05).

The radial growth of *A. alternata* was reduced by 60.5%; *C. luteo-olivacea* by 54.76%; *D. macrodydima* by 44.7%; *D. seriata* by 36.4%; and *F. fujikuroi* by 41.7%. In the case of *F. oxysporum*, one of the most ubiquitous fungal phytopathogens, *Streptomyces* sp. HR58 was able to reduce fungal growth by almost 50%. The growth of *P. chlamydospora*, *P. richardsiae* and *R. solani* was reduced by 46.5%, 61.8% and 68%, respectively. Different *V. dahliae* strains showed different degrees of inhibition. In the case of the most aggressive pathogenic strain (V937I, isolated from olive trees), growth inhibition reached 65.7%. In strains isolated from sunflowers, however, inhibition values ranged from 78.2% for the defoliating strain (D0913) to 80.9% for the non-defoliating strain (ND1113), as represented in Figure 4.

### 3.5. Purification and Identification of Cycloheximide Production

The antiSMASH analysis revealed a putative cycloheximide BGC in the genome of strain HR58. These data suggest that the observed antifungal activity could be attributed, at least in part, to the production of this compound.

To evaluate the putative biosynthesis of this antifungal compound, liquid cultures were established, and culture supernatants were analysed by HPLC. Chromatographic analysis of the HR58 extract revealed a peak at 6.93 min, coinciding with the retention time of the commercial cycloheximide standard (Figure 5A). The UV absorption spectrum of this peak showed a profile comparable to that of the standard, with an absorption maximum at approximately 210–220 nm (Figure 5B). These results support the identification of a cycloheximide-like compound in the HR58 extract.

The fraction corresponding to the peak of interest was collected in 30 separate runs. Subsequently, it was extracted with ethyl acetate, and the residue was dissolved in 85 µL of 80% methanol. An antifungal activity test was performed using a plate bioassay. The fraction exhibited clear growth inhibition of *V. dahliae*, supporting the antifungal activity of the collected fraction (Figure 6).

### 3.6. Synthesis and Characterization of AgNPs

The synthesis of silver nanoparticles (AgNPs) by the cell-free filtrate of *Streptomyces* sp. HR58 was monitored over a 72-h incubation period through visual inspection and UV–visible spectroscopy at 24 h intervals. A progressive change in the reaction mixture from colorless to a yellowish-brown coloration was observed, indicating the reduction of silver ions and the formation of AgNPs. In contrast, no visible color change was observed in the control samples containing either AgNO_3_ or the cell-free filtrate alone.

The UV-visible spectra corroborated these observations (Figure 7). At the beginning of the experiment (0 h), no characteristic absorption peak associated with AgNP formation was detected in any of the reaction mixtures, including the controls and the AgNO_3_ solution supplemented with the cell-free filtrate. After 72 h of incubation, however, the AgNO_3_-treated cell-free filtrate exhibited a broad surface plasmon resonance (SPR) band with a maximum between 410 and 420 nm, confirming the successful biosynthesis of AgNPs. No color change was observed in any of the controls, including ISP2 supplemented with AgNO_3_, the HR58 cell-free filtrate, or AgNO_3_ alone, either at the beginning of the experiment or after 72 h (Supplementary Material Figure S2).

Morphological analysis of the synthesized biogenic AgNPs, performed using transmission electron microscopy, revealed their morphology and size (Figure 8). The TEM micrographs demonstrated the relatively spherical shape of the AgNPs and their tendency to aggregate. The nanoparticles had an average size of 23.65 nm, with a standard deviation of 4.26 nm.

### 3.7. Antibiotic Activity of AgNPs

The antibiotic activity of AgNPs synthesized from the cell-free supernatant of *Streptomyces* sp. HR58 was measured using the well-diffusion method (Table 4). Inhibition was observed against *Bacillus subtilis*, *Enterococcus faecalis* and *Salmonella typhimurium,* whereas no inhibition was observed against *Escherichia coli* or the yeasts (*Candida albicans* and *Saccharomyces cerevisiae*). AgNPs were found to inhibit the growth of fungal phytopathogens *Alternaria alternata*, *Fusarium fujikuroi*, *F. oxysporum* and *Verticillium dahlia*, producing inhibition halos of 10.81, 2.62, 3.40 and 14.00 mm, respectively.

The best results were obtained for *V. dahliae*, reaching an inhibition halo of 15 ± 4.0 mm with 190 µg of HR58 AgNPs. No inhibition was observed at 0.5 or 1 µg of HR58 AgNPs (Figure 9). A dose-dependent increase in the inhibition of *V. dahliae* was observed, with the largest inhibition zone at 190 µg. The overall effect of AgNP dose was significant (Kruskal–Wallis H = 19.37, *p* = 0.0036). After Dunn’s test with Holm correction, only the 190-µg dose differed significantly from the water control (adjusted *p* = 0.0089), whereas none of the AgNP doses differed significantly from the 1 mM AgNO_3_ control (Table S5).

## 4. Discussion

Several rhizobacteria have been reported as biological control agents (BCAs), with *Streptomyces* being one of the most important genera because of its prolific production of bioactive compounds [41]. Identifying *Streptomyces* species represents a major challenge due to the complexity of their taxonomy and the large number of species within the genus, which is one of the largest groups in the domain Bacteria, with more than 810 validly named species (https://lpsn.dsmz.de/genus/streptomyces, accessed on 7 May 2026). In this study, we identified and characterised the strain *Streptomyces* sp. HR58, a promising biocontrol agent isolated from the hop rhizosphere.

Morphological traits such as spore colour, hyphal morphology, pigment production and the utilisation of different sugars as carbon sources in physiological assays are considered consistent and reliable characteristics for taxonomic classification. The International Streptomyces Project (ISP) published descriptions of type strains for 458 *Streptomyces* species [17]. Phenotypic characterisation of *Streptomyces* sp. HR58 revealed a characteristic pigmentation with colours close to papyrus white, which have also been reported in other species such as *S. shenzhenensis* [42] and *S. albiflaviniger* [43]. The growth pattern of the strain across different temperature conditions indicates a preference for low to moderate temperatures. In contrast, it tolerated a wide pH range in DSMZ 65 medium. As reported by Kontro et al. [44], growth media containing complex nutrients such as yeast extract may support a wider pH tolerance in *Streptomyces*.

At the genomic level, long-read sequencing revealed that HR58 has a single-contig genome of 8.27 Mb with a GC content of 71.76%, consistent with the large, high-GC genomes typically observed in the genus *Streptomyces* [45]. The recovery of a complete genome provides a robust framework for comparative genomics and genome mining analyses, minimizing fragmentation-associated uncertainties in biosynthetic gene cluster prediction.

Genome-based phylogenomic analyses placed HR58 within the *Streptomyces anulatus* clade. ANI calculations revealed 96.6% nucleotide identity with *S. anulatus*, whereas the dDDH value was 66.5%, remaining slightly below the classical 70% species delineation threshold [29]. Although ANI values above 95% are commonly considered compatible with species-level assignment [27], recent studies have shown that, within the genus *Streptomyces*, a 70% dDDH value corresponds more closely to approximately 96.7% ANI rather than the general bacterial ANI threshold of 95% [42]. Moreover, these authors proposed that strains displaying genome-relatedness values close to these cut-offs should be interpreted using an integrated taxonomic framework combining phylogenomics, phenotypic characterisation and chemotaxonomic evidence.

Therefore, although HR58 is clearly affiliated with the *S. anulatus* lineage, the slight discordance observed between ANI and dDDH values suggests that the strain should be conservatively regarded as belonging to the *S. anulatus* species complex/clade rather than being unequivocally assigned to this species. Similar borderline genome relatedness values have been reported in other closely related *Streptomyces* taxa and likely reflect the complex evolutionary history and extensive genomic conservation characteristic of this genus.

AntiSMASH analysis of the *Streptomyces* HR58 genome identified 35 BGCs, including NRPS, PKS, hybrid NRPS–PKS systems, RiPPs, terpenes and siderophores, with 14 clusters showing more than 75% similarity to characterized BGCs. The presence of four cryptic clusters without detectable similarity to known BGCs in HR58 suggests that this strain may harbor additional biosynthetic potential that remains unexplored under specific environmental conditions [46]. This is consistent with studies showing that biotic interactions between *Streptomyces* and fungi can induce the expression of otherwise silent BGCs [5,47].

Comparative analysis with *S. anulatus* ATCC 11523 indicated that the specialized-metabolite repertoires of the two strains were broadly similar. The reference strain contained 36 predicted BGCs, only one more than the 35 regions identified in HR58, with extensive conservation of the main BGC classes and several known-cluster assignments. Nevertheless, differences in the total number of antiSMASH regions should be interpreted cautiously because adjacent biosynthetic loci may be merged or split according to their predicted boundaries. The clearest locus-level difference was an actinomycin D-related region in ATCC 11523 [40], which was absent from the HR58 genome, whereas the corresponding interval in HR58 contained a distinct NRPS/PKS-associated region. However, the presence or absence of a predicted BGC does not demonstrate metabolite production, and this difference cannot be directly associated with the observed antifungal phenotype without expression or chemical evidence.

Among the predicted antifungal-related pathways in HR58, a trans-AT PKS region showing high similarity (94%) to the cycloheximide biosynthetic gene cluster was detected. Cycloheximide is a well-known glutarimide antibiotic that inhibits eukaryotic protein synthesis at the elongation step [48] and has been implicated in the antifungal activity of several *Streptomyces* strains used as BCAs [49,50]. In *Streptomyces* sp. HR58, this genomic prediction was corroborated by HPLC–DAD analysis and bioassays against *V. dahlia*. The HR58 extract showed a peak with the same retention time and UV absorption profile as a commercial cycloheximide standard, and the corresponding collected fraction displayed antifungal activity. Together, these findings provide strong evidence for the production of a cycloheximide-like antifungal compound by HR58. However, definitive structural confirmation would require complementary LC-MS/MS or NMR-based analyses. Furthermore, because the HR58 genome harbours several additional biosynthetic gene clusters with potential antifungal activity, as well as genes encoding putative cell wall-degrading enzymes, the observed antagonistic phenotype cannot be attributed exclusively to cycloheximide. Therefore, cycloheximide should be regarded as a major, but not exclusive, contributor to the antifungal activity of HR58. Further chemical and genetic studies will be necessary to determine the relative contribution of cycloheximide and other antifungal mechanisms to fungal inhibition by this strain.

Cycloheximide should therefore be considered a plausible contributor to the antifungal activity of HR58, potentially an important one, but not its exclusive determinant. Other predicted specialized metabolites, together with the repertoire of hydrolytic enzymes encoded by the genome, may also contribute to the observed antagonistic phenotype. Moreover, because cycloheximide inhibits eukaryotic translation, its production raises biosafety and regulatory concerns regarding the direct release of HR58 into agricultural environments. Any future application of this strain would require quantification of cycloheximide production and persistence, assessment of potential effects on non-target organisms, and the development of appropriate containment or formulation strategies.

The identification of a cycloheximide biosynthetic gene cluster (BGC) in the genome of strain HR58 may contribute to its antifungal potential, although the presence of this BGC does not necessarily imply that cycloheximide is produced under environmental conditions. As previously discussed for *Streptomyces*-based biocontrol agents, antimicrobial secondary metabolites constitute an important mechanism of antagonism against phytopathogens and are naturally involved in localized interactions between microorganisms in the soil [51]. If expressed by HR58 under environmental conditions, cycloheximide production would be expected to occur locally in the immediate vicinity of the producing cells, where concentrations may be sufficient to affect neighbouring microorganisms while remaining localized around the producing cells, thereby limiting exposure beyond the immediate microbial community. Thus, the potential exposure of non-target organisms, including humans and animals, would be expected to be limited. Nevertheless, further studies are required to confirm cycloheximide production by HR58 under environmentally relevant conditions and to determine its concentration, persistence, and potential effects on non-target soil microorganisms before the strain can be considered for practical agricultural applications.

Beyond antibiotic production, our genomic analysis suggests that HR58 encodes an extensive repertoire of enzymes that may contribute to fungal cell wall degradation, including eight chitinases, four chitin-binding proteins, two β-N-acetylglucosaminidases, four endo-β-1,4-glucanases, and at least 62 proteases, many of which are predicted to be secreted. Similar combinations of chitinolytic and proteolytic enzymes have been associated with the degradation of fungal cell walls and microsclerotia, enhancing the biocontrol efficacy of *Streptomyces* and other antagonistic bacteria under both in vitro and in planta conditions [52,53,54]. HR58 may combine specialized metabolite production with hydrolytic activities potentially targeting structural components of the fungal cell wall. Such multi-component modes of action are considered advantageous for durable biocontrol, as they may decrease the risk of resistance development compared with single-target fungicides [55].

The functional annotation also identified traits frequently associated with plant-associated bacteria, including putative ACC deaminases, extracellular and broad-specificity phosphatases and siderophore-related functions (Table S6). Collectively, these features suggest potential capacities for modulation of plant stress responses, phosphorus acquisition and competition for iron in the rhizosphere. However, most of these functional categories were also represented in the reference strain *S. anulatus* ATCC 11523 and cannot be considered unique features of HR58. Furthermore, HR58 cannot yet be described as a plant growth-promoting rhizobacterium based on genomic evidence alone, because ACC deaminase activity, phosphate mobilization, siderophore production and plant growth promotion were not experimentally assessed.

*Streptomyces* spp. are consistently referred to in the literature as potential BCAs against a large number of fungal pathogens, including *Alternaria* [56], *Cadophora* [57], *Dactylonectria* [58], *Diplodia* [59], *Fusarium* [60,61], *Phaeomoniella* [58,62], *Rhizoctonia* [63] or *Verticillium* [33]. However, to the best of our knowledge, no studies have investigated the antifungal activity of *Streptomyces* against *Pleurostoma* or *Thelonectria.* In our study, we assessed the activity of the strain HR58 against 13 different isolates of fungal phytopathogens affecting different crops. Our findings indicate that *Streptomyces* sp. HR58 exhibits strong antifungal activity against *Alternaria alternata*, *Pleurostoma richardsiae*, *Rhizoctonia solani* and *Verticillium dahliae,* with inhibition indices over 60%. Díaz-Díaz et al. [64] reported that six *Streptomyces* strains evaluated against *V. dahliae* showed moderate mycelial growth inhibition in dual culture assays, ranging from 33.6% to 44.4% (against isolates V323 (D pathotype) and V004 (ND pathotype), whereas *Streptomyces* sp. HR58 exhibited an inhibition index of 80% against *V. dahliae* (both D and ND pathotypes), indicating that antagonistic performance within this genus can depend on the strain and the target structure.

In addition to its biocontrol traits, *Streptomyces* HR58 demonstrated the ability to mediate the extracellular biosynthesis of silver nanoparticles (AgNPs) using cell-free culture filtrates. The resulting AgNPs displayed a characteristic surface plasmon resonance band between 410 and 420 nm and an average particle size of approximately 24 nm with a predominantly spherical morphology. Similar results have been reported for *S. hygroscopicus* [13] with a SPR band at 420–425 nm and an average size of 20–30 nm, and for *S. griseoplanus* [12] with a SPR band at 413–417 nm and a particle diameter ranging from 19.5–20.9 nm. Although numerous studies have reported broad-spectrum antimicrobial activity for silver nanoparticles (AgNPs) synthesized by *Streptomyces* spp., the AgNPs produced by strain HR58 displayed a more restricted antimicrobial profile. In our study, significant antifungal activity was only observed against *Alternaria alternata*, *Fusarium fujikuroi*, *F. oxysporum* and *Verticillium dahliae*. In contrast, no significant inhibitory effects were detected against the yeast or bacterial strains tested, although a slight antibacterial activity was observed against *Bacillus subtilis*, *Enterococcus faecalis* and *Salmonella typhimurium*. These findings differ from previous reports describing pronounced antibacterial activity of *Streptomyces*-derived AgNPs, suggesting that the antimicrobial spectrum of biologically synthesized nanoparticles may vary depending on factors such as the producing strain, nanoparticle physicochemical properties, or the experimental conditions employed [8,14,65]. It is known that the release of silver ions following the interaction between AgNPs and the fungal cell surface disrupts cell replication, intracellular homeostasis and induces hyphal vacuolation, leading to cell death [66]. A plausible explanation is that the size, surface chemistry and capping biomolecules associated with the HR58 secretome-derived nanoparticles may confer a certain degree of specificity toward fungal hyphae, likely due to differences in cell wall composition. However, this hypothesis requires further physicochemical characterisation.

The genomic survey identified several redox-related protein families in HR58, including NAD(P)H-dependent flavin oxidoreductases, ferric-siderophore reductases, nitroreductase-family proteins and thioredoxin-related proteins (Table S6). These functions are compatible with a metabolically active redox network and provide plausible candidates for processes that may contribute indirectly to the generation of reducing or capping components in the extracellular medium. Nevertheless, comparable functional categories were also abundant in *S. anulatus* ATCC 11523, and none of the identified proteins can be specifically assigned to Ag(I) reduction. The genomic data therefore do not demonstrate the existence of a dedicated AgNP biosynthetic pathway or identify the extracellular component responsible for nanoparticle formation. Proteomic or metabolomic characterization of the cell-free filtrate, combined with fractionation and controlled Ag(I)-reduction assays, would be required to investigate this mechanism. In addition, the AgNP experiments should be interpreted as a separate cell-free application of the HR58 secretome rather than as a direct property of the living biocontrol agent. Further characterization of nanoparticle stability, surface chemistry and effects on non-target organisms would also be necessary before considering agricultural applications.

The dual capacity of HR58 to function as a biocontrol agent and as a biological factory for AgNPs opens promising avenues for integrated strategies that combine microbial inoculants with nanotechnology-based tools in plant disease management. Future studies should therefore focus on testing HR58 in greenhouse and field trials on susceptible crops, both as a free-living rhizosphere inoculant and, potentially, as part of bioformulations tailored for fungal-infested soils. Combining RNA-seq and untargeted metabolomics under co-culture conditions with fungal pathogens could help elucidate which BGCs and hydrolytic enzymes are expressed in planta and how they contribute to disease suppression. Together, these efforts would enable a more comprehensive assessment of *Streptomyces* HR58 as a versatile component of sustainable, multi-modal strategies for the management of Verticillium wilt and other soil-borne diseases.

## 5. Conclusions

In summary, *Streptomyces* sp. HR58 represents a promising actinobacterial strain with considerable potential as a biological control agent against fungal phytopathogens. Its remarkable physiological adaptability—evidenced by its ability to grow over a wide temperature range (4–30 °C), across a wide pH range (5–12), and tolerate salinity of up to 5% NaCl—is further supported by its genomic and biosynthetic potential, including the presence of a cycloheximide biosynthetic gene cluster and the demonstrated production of cycloheximide in liquid cultures. Moreover, the ability of HR58 to biosynthesize antifungal silver nanoparticles provides an additional biotechnological trait that may contribute to its effectiveness against plant pathogens. The ANI and dDDH results also highlight the taxonomic complexity of the genus *Streptomyces*, as HR58 could not be confidently assigned to any currently described species. Overall, these findings support *Streptomyces* sp. HR58 as a versatile and promising candidate for the development of sustainable strategies for plant disease management.

## Figures and Tables

**Figure 1:**
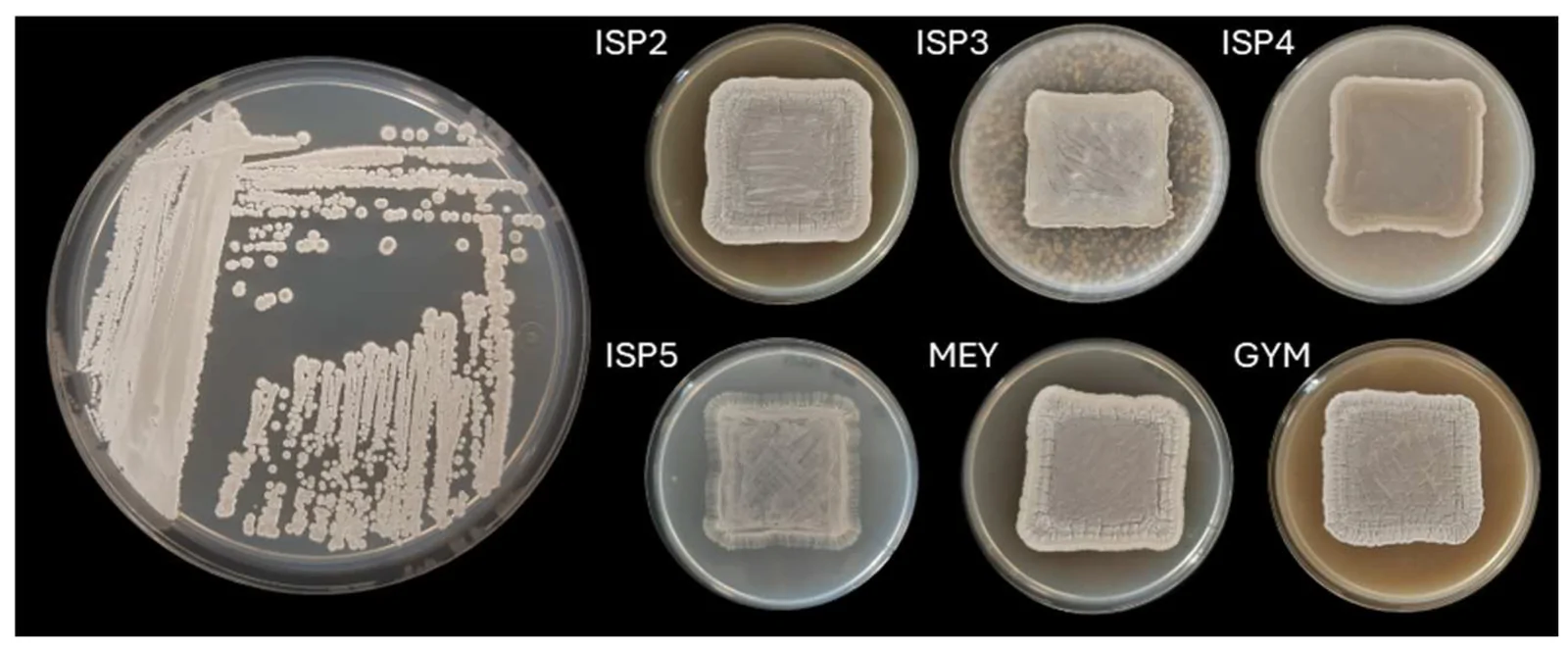
Cultural and morphological properties of HR58 on MEY (left plate) and several culture media after 7 days at 28 °C.

**Figure 2:**
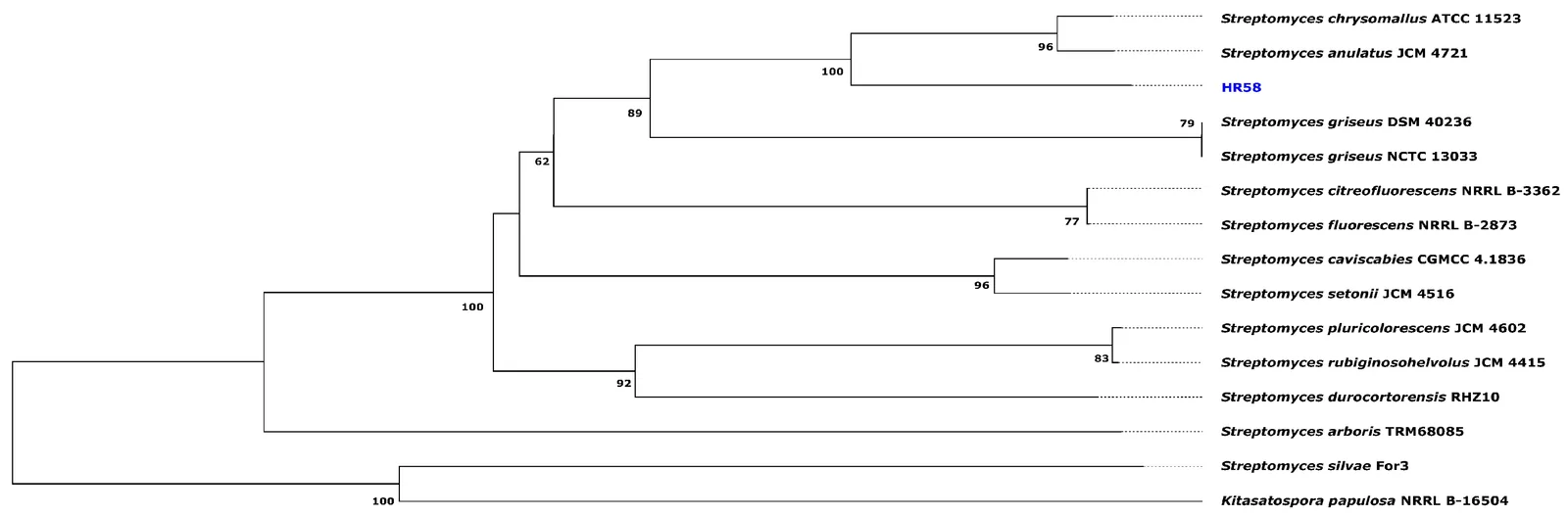
Whole-genome phylogenomic tree inferred using TYGS based on Genome BLAST Distance Phylogeny (GBDP) distances. Numbers at nodes indicate pseudo-bootstrap support values.

**Figure 3:**
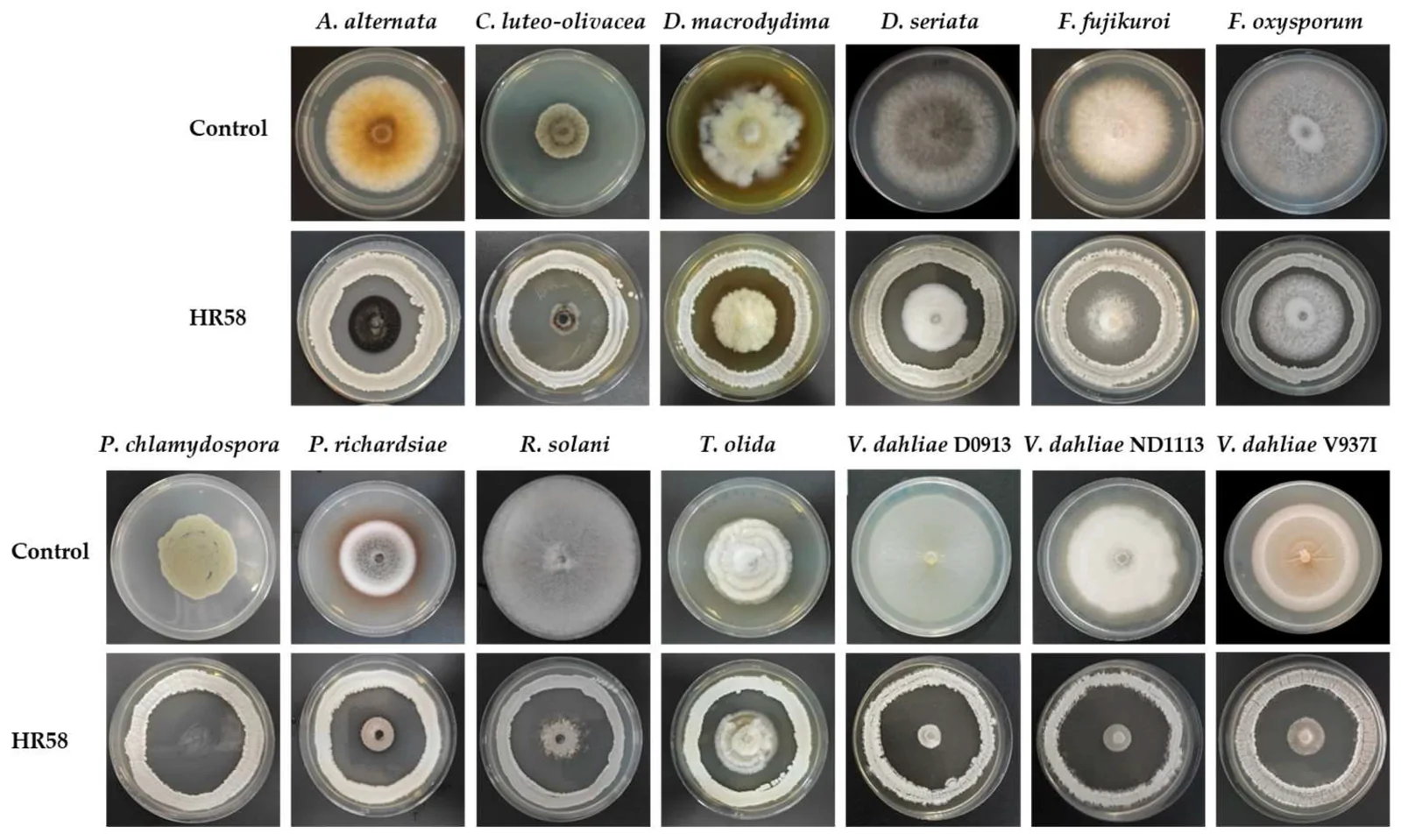
Antifungal activity of *Streptomyces* sp. HR58 against different phytopathogenic fungi. For each fungus tested, the upper image corresponds to the growth control, while the lower image shows fungal growth in the presence of *Streptomyces* sp. HR58.

**Figure 4:**
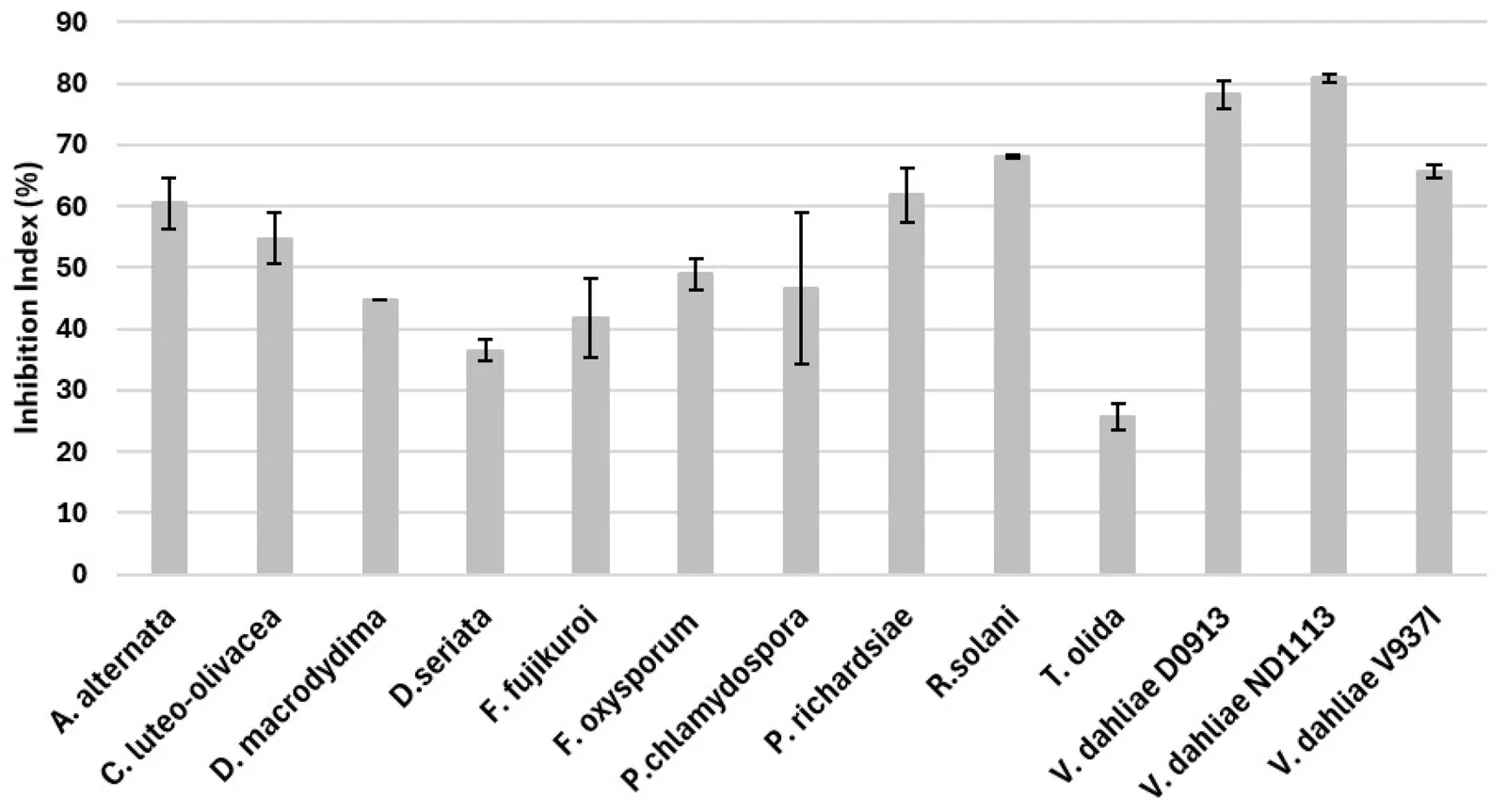
Quantification of antifungal activity of *Streptomyces* sp. HR58 against different fungal phytopathogens based on the calculation of the Inhibition index (I-index). Error bars represent the standard deviation (SD) of three independent replicates.

**Figure 5:**
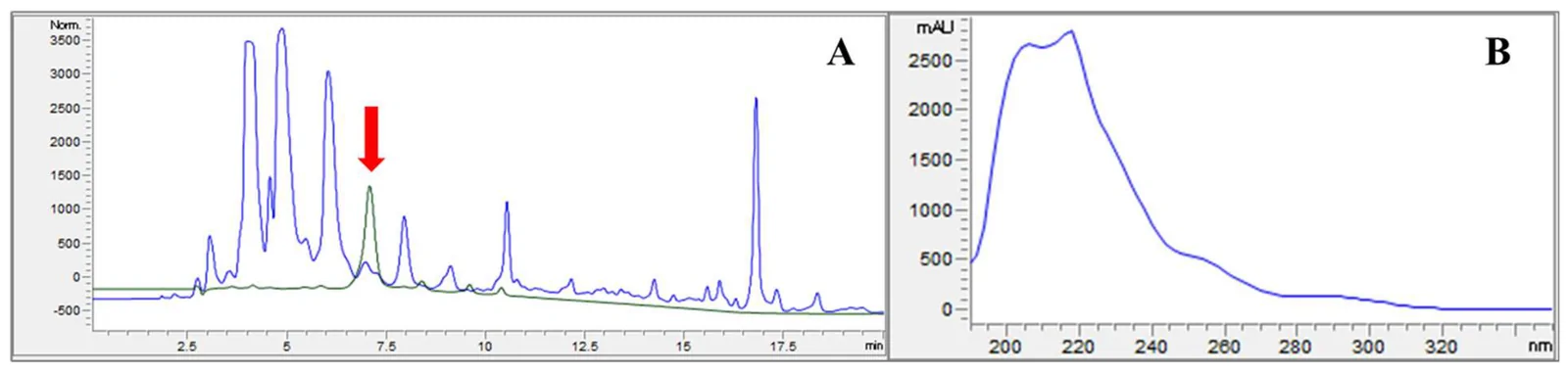
(**A**) HPLC chromatograms of a 10 mg/ml cycloheximide standard (green trace) and HR58 extract (blue trace); The red arrow indicates the peak corresponding to the retention time of commercial chlorhexidine. (**B**) UV spectrum of cycloheximide.

**Figure 6:**
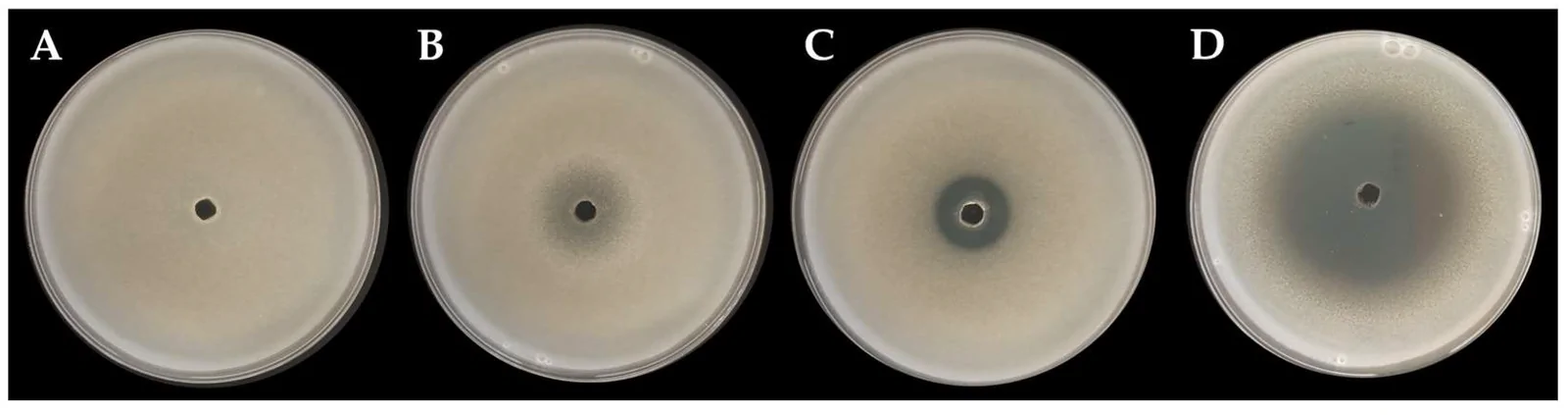
Antifungal activity against *V. dahliae* of 60 μL of: (**A**) 80% methanol (solvent control); (**B**) an HPLC fraction collected at a retention time of 6.93 min from the culture supernatant of strain HR58 grown in liquid medium; (**C**) the same HPLC fraction concentrated 30-fold; (**D**) commercial cycloheximide (10 mg/mL, positive control).

**Figure 7:**
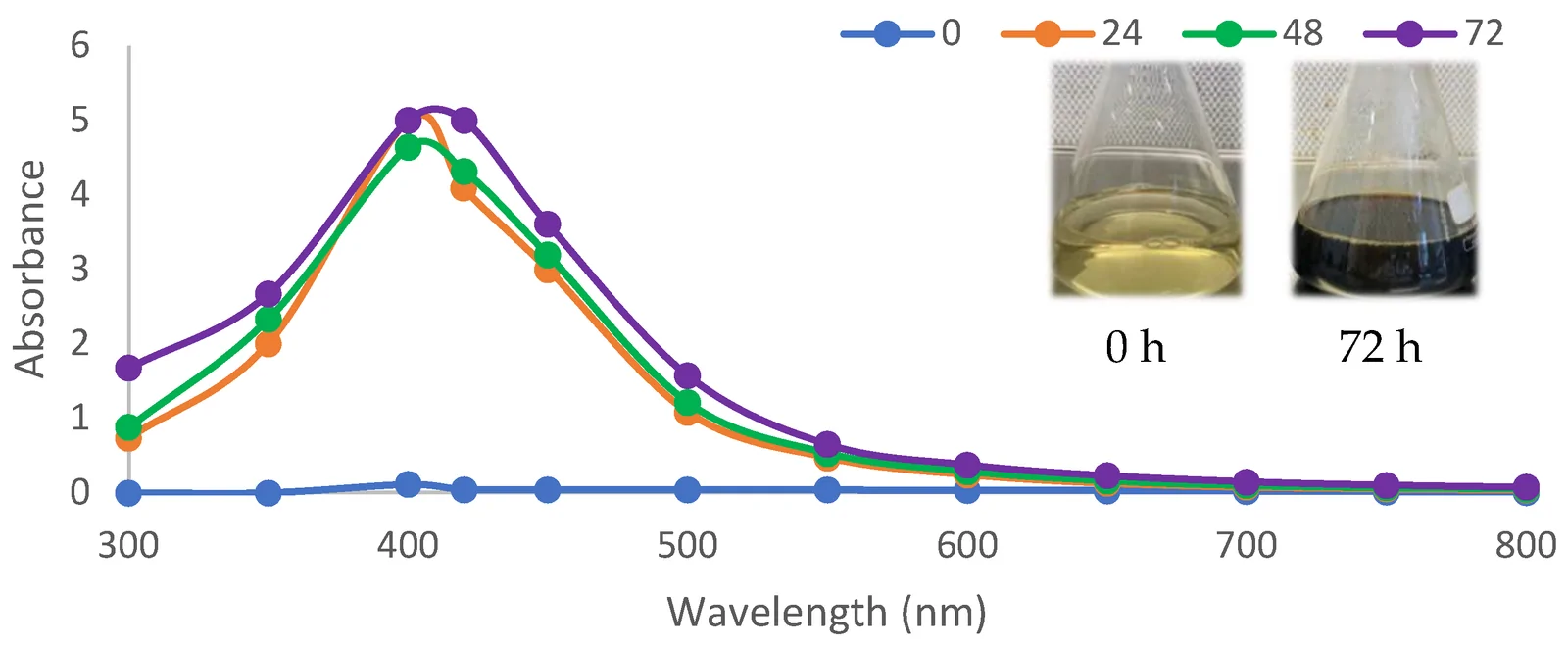
UV–visible spectroscopy of biogenic AgNPs formed from the extracellular extracts of the *Streptomyces* sp. HR58 strain.

**Figure 8:**
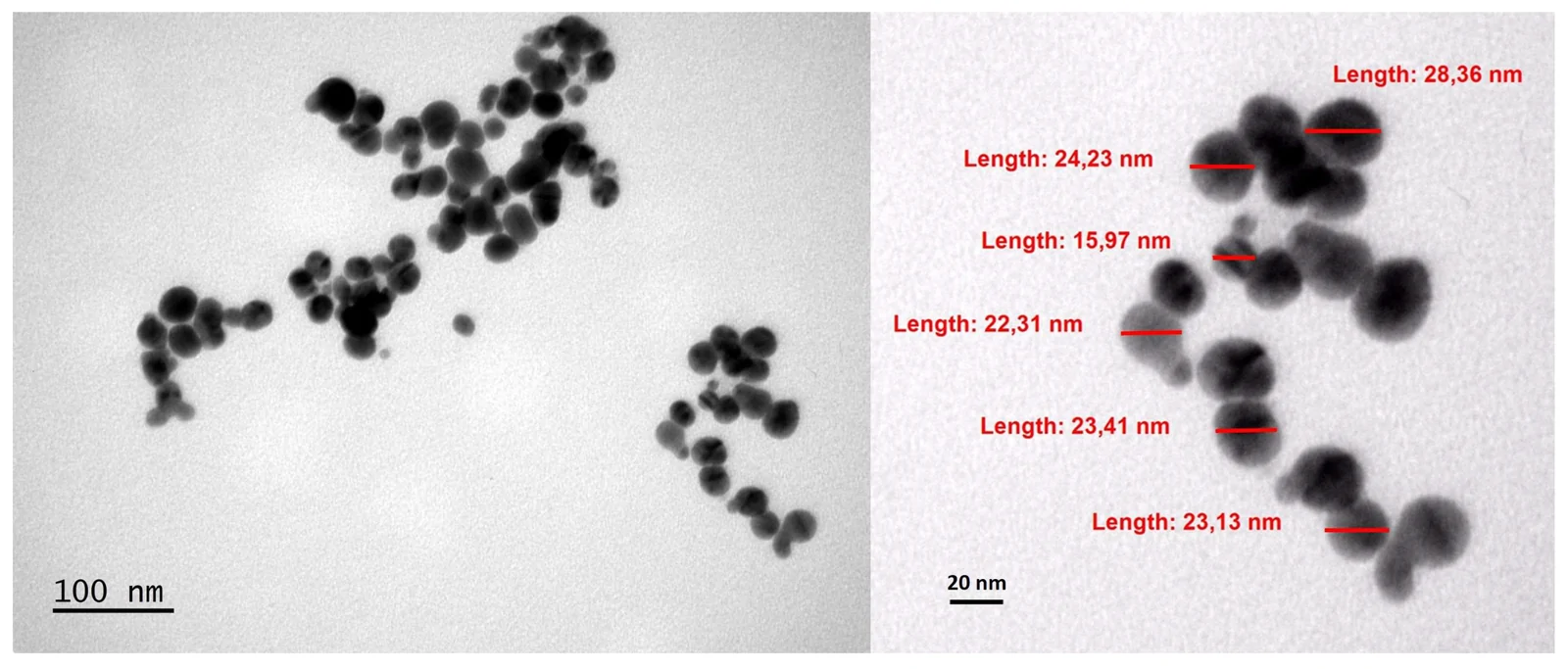
Transmission electron microscopy image of biogenic AgNPs formed from the extracellular extracts of the *Streptomyces* sp. HR58 strain.

**Figure 9:**
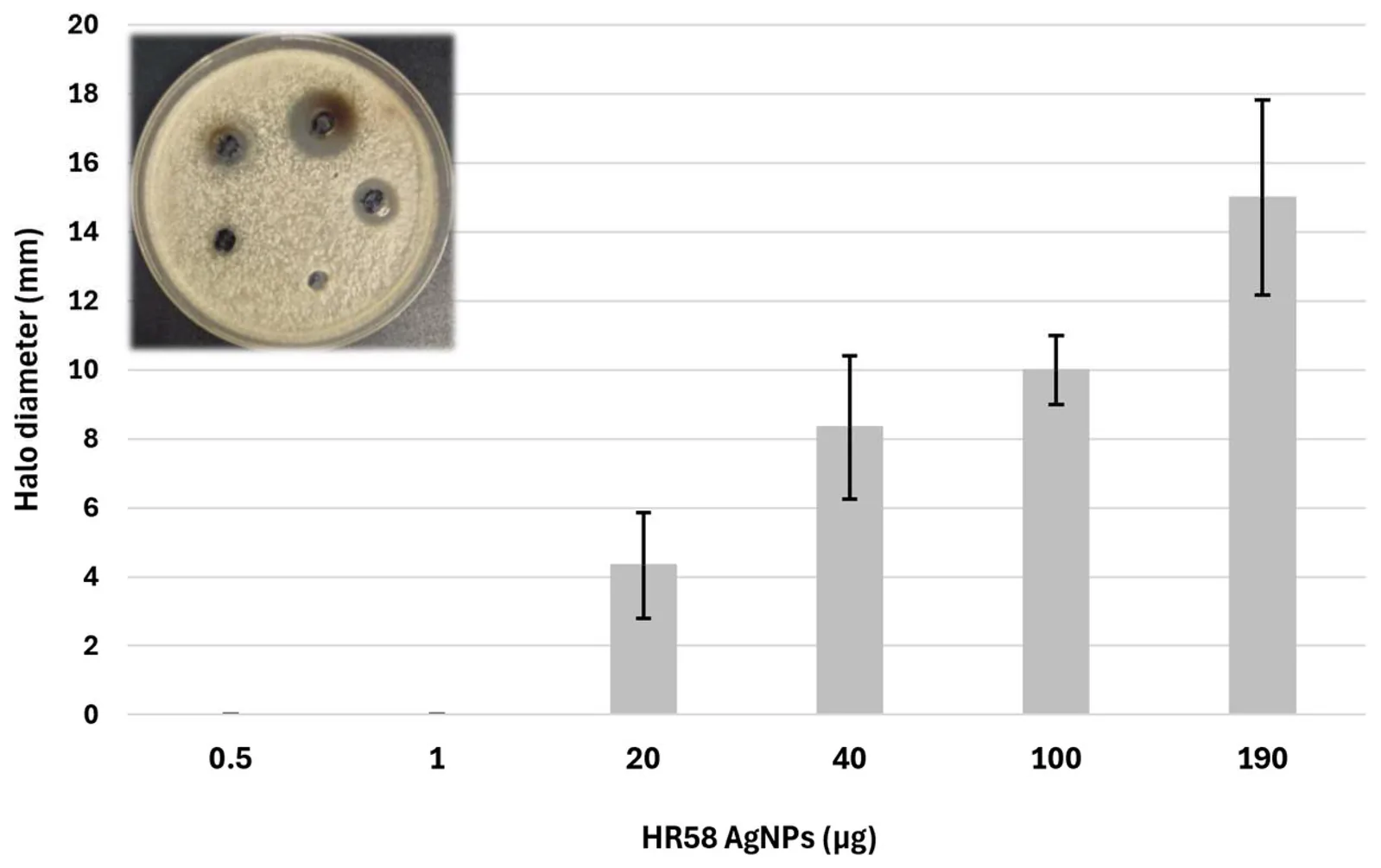
Antifungal activity of *Streptomyces* sp. HR58 AgNPs against *V. dahliae*.

**Table 1:** Genome features of *Streptomyces* sp. HR58.

Feature	Value
Number of contigs	1
Genome size (bp)	8,270,372
GC content (%)	71.76
Protein-coding sequences (CDS)	7299
RNA genes	84
Functional subsystems (RAST)	306
Biosynthetic gene clusters (BGCs)	35

**Table 2:** Distribution of biosynthetic gene clusters in *Streptomyces* sp. HR58 according to similarity with known clusters.

BGC Similarity to Known Clusters	Number of BGCs
100% similarity	10
75–99% similarity	4
30–70% similarity	3
1–29% similarity	14
No detectable similarity (cryptic)	4
Total	35

**Table 3:** Enzymes associated with fungal cell wall degradation in *Streptomyces* sp. HR58.

Enzyme Group	Number of Genes	Relevant Features
Chitinases	8	7 predicted secreted (SignalP-6.0)
Chitin-binding proteins	4	Putative CBPs involved in substrate recognition
β-N-acetylglucosaminidases	2	Terminal enzymes of chitin degradation
Endo-1,4-β-glucanases	4	Putative glucan-degrading enzymes
Proteases	62	23 predicted secreted; 38 > 1000 bp

**Table 4:** Antibiotic activity of *Streptomyces* sp. HR58 AgNPs (190 µg).

Microorganisms	Type	Halo (mm)
*Bacillus subtilis*	Gram +	6.85 ± 1.62
*Enterococcus faecalis*	Gram +	8.75 ± 1.06
*Escherichia coli*	Gram −	0
*Salmonella typhimurium*	Gram −	7.75 ± 2.47
*Candida utilis*		0
*Saccharomyces cerevisiae*		0
*Alternaria alternata*		10.81 ± 1.42
*Fusarium fujikuroi*		2.62 ± 0.38
*Fusarium oxysporum*		3.4 ± 0.73
*Verticillium dahliae*		15 ± 4.00

## Data Availability

The genome sequences of strainHR58 have been deposited in the GenBank database under the accession number SUB15943355. The associated BioProject accession number is PRJNA1230872.

## Supplementary Materials

The following supporting information can be downloaded at https://www.mdpi.com/article/10.3390/microorganisms14102344/s1. Figure S1: Antifungal activity of *Streptomyces lacaronensis* OE57 against different fungal phytopathogens; Figure S2: Appearance of the samples at the beginning of the experiment (0 h) and after 72 h of incubation; Figure S3: Functional classification of genes identified in *S. anulatus* ATCC 11523 and HR58 according to their predicted metabolic and cellular functions; Table S1: Phenotypic properties of *Streptomyces* sp. HR58; Table S2: Biochemical and enzymatic properties of the strain HR58 according to API 20NE and API-ZYM tests; Table S3: Biosynthetic gene clusters (BGCs) predicted by AntiSMASH v 7.1 in the *Streptomyces* sp. HR58 genome. Table S4: Descriptive and inferential statistical analysis of fungal growth inhibition by HR58 and the positive-control strain *S. lacaronensis* OE57; Table S5: Descriptive and inferential statistical analysis of inhibition-zone diameters against *Verticillium dahliae* in the HR58-derived AgNP dose–response assay; Table S6: Putative genomic traits of *Streptomyces* sp. HR58 potentially associated with plant growth promotion, antifungal activity and extracellular AgNPs formation.
