# Supplementary material for: Phenotypic and Genomic Characterization of Streptomyces sp. HR58 Reveals Its Potential Against Fungal Phytopathogens
Source: Microorganisms. 2026 Oct 14;14(10):2344. doi: 10.3390/microorganisms14102344 (PMC13707951; doi:10.3390/microorganisms14102344)
Supplement: Supplementary file 1 [file microorganisms-14-02344-s001.zip › microorganisms-4472619-supplementary.pdf]

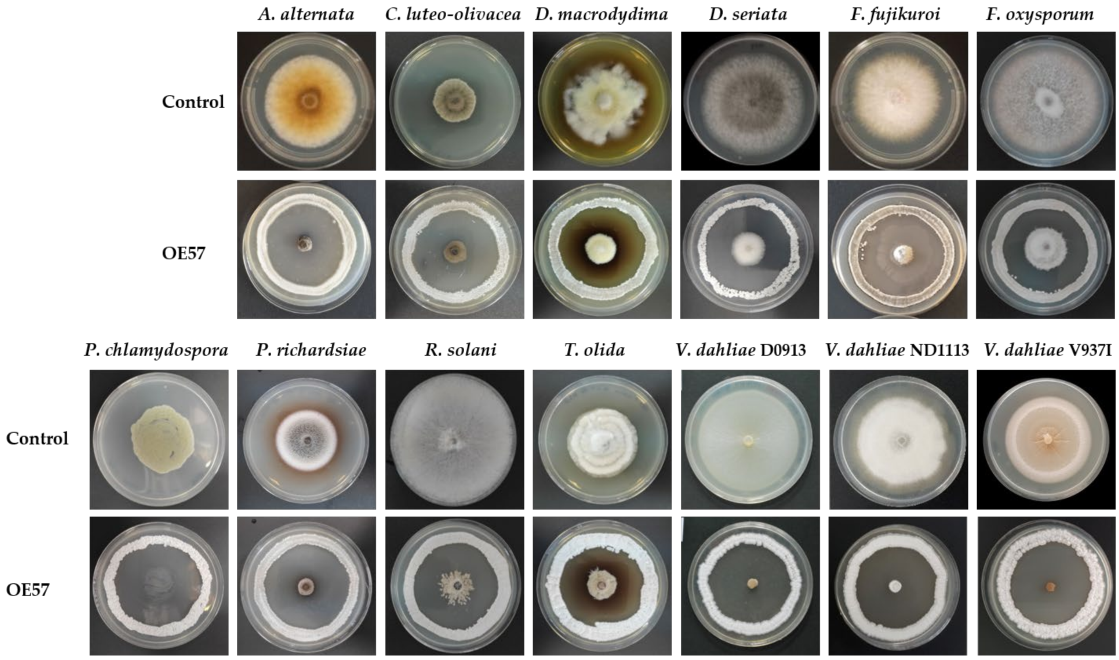

Figure S1. Antifungal activity of *Streptomyces lacaronensis* OE57 against different fungal phytopathogens. The upper row shows the corresponding fungal controls, whereas the lower row shows fungal growth in the presence of strain OE57.

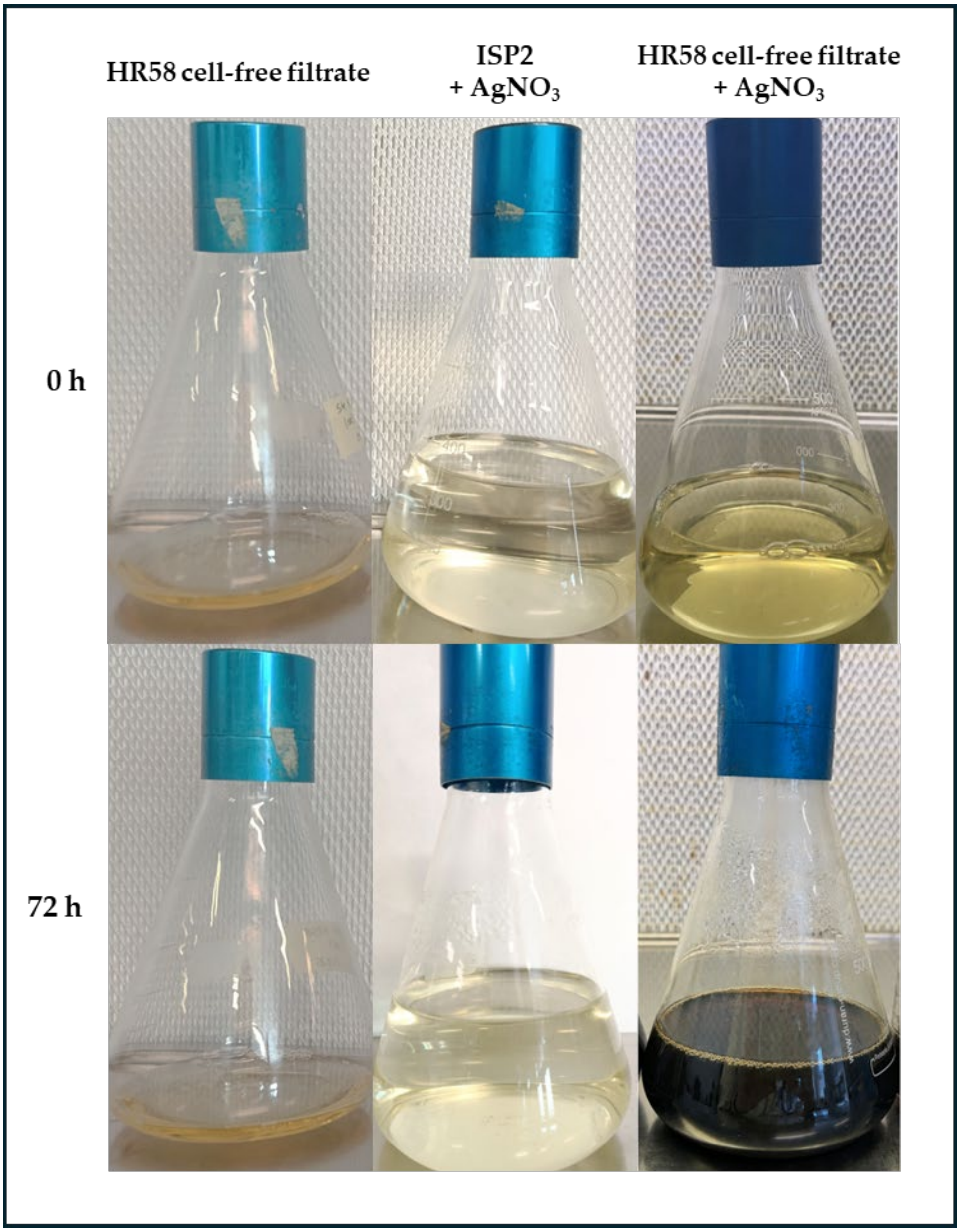

Figure S2. Appearance of the samples at at the beginning of the experiment (0 h) and after 72 h of incubation: left, HR58 culture supernatant; centre, ISP2 culture medium supplemented with $AgNO_3$ (control); and right, HR58 culture supernatant supplemented with $AgNO_3$.

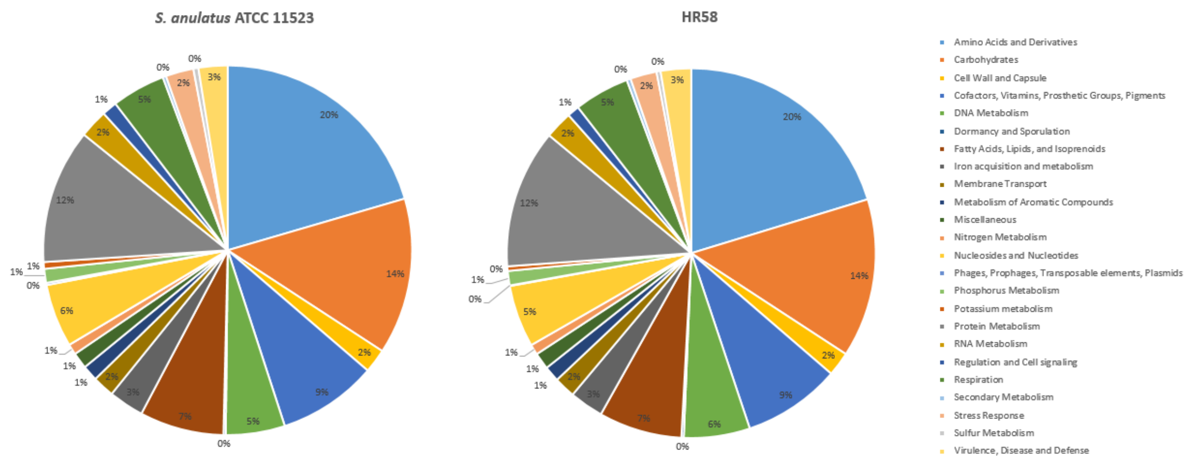

Figure S3. Functional classification of genes identified in *S. anulatus* ATCC 11523 and HR58 according to their predicted metabolic and cellular functions. The pie charts show the relative distribution of genes among the different functional categories.

Table S1. Phenotypic properties of *Streptomyces* sp. HR58 (+ weak growth; ++ moderate growth; +++ good growth)

Temperature tests		Tolerance tests		Agar media tests		
ºC	Growth	pH	Growth	Medium	Growth	Description (RAL colour codes)
4	+	5.0	+++	ISP1	++	Grey white aerial mycelium (9002). Cream susbtrate mycelium (9001)
10	++	5.5	+++	ISP2	+++	Papyrus white aerial mycelium (9018). Ochre yellow substrate mycelium (1024)
15	++	6.0	+++	ISP3	+++	Green beige aerial mycelium (1000). Cream substrate mycelium (9001)
20	+++	7.5	+++	ISP4	+	Papyrus white aerial mycelium (9018). Cream substrate mycelium (9001)
25	+++	8.0	+++	ISP5	+	Oyster white aerial mycelium (1013). Cream substrate mycelium (9001)
28	+++	8.5	+++	ISP6	+++	Beige red substrate mycelium (3012)
30	+++	10.0	+++	ISP7	++	Light grey aerial mycelium (7035). Beige substrate mycelium (1001) with salmon range diffusible pigment (2012)
37	-	12.0	+++	TSA	+	Brown beige substrate mycelium (1011)
42	-	NaCl	Growth	NA	++	Signal white aerial mycelium (9003). Cream substrate mycelium (9001)
45	-	2.5%	+++	Bennet	+++	Grey white aerial mycelium (9002). Curry substrate mycelium (1027)
		5.0%	++	R5	+++	Papyrus white aerial mycelium (9018). Curry substrate mycelium (1027)
		7.5%	-	GYM	+++	Papyrus white aerial mycelium (9018). Ochre yellow substrate mycelium (1024)

Table S2. Biochemical and enzymatic properties of the strain HR58 according to API 20NE and API-ZYM tests.

API 20NE		API ZYM		
**Reaction/Enzyme**	**Results**	**Nº**	**Enzyme**	**Results**
Nitrate/nitrite reductase	-	1	Control	-
Tryptophanase	-	2	Alkaline Phosphatase	+
Glucose fermentation	-	3	Esterase (C 4)	+
Arginine dihydrolase	-	4	Esterase lipase (C 8)	+
Urease	+	5	Lipase (C 14)	+
Hydrolysis (esculin)	+	6	Leucine arylamidase	+
Hydrolysis (gelatine)	+	7	Valine arylamidase	+
p-Nitro-phenyl-β-D-galactopyranosidase	+	8	Cystine arylamidase	+
Glucose assimilation	+	9	Trypsin	+
Arabinose assimilation	+	10	α-chymotrypsin	+
Mannose assimilation	+	11	Acid phosphatase	+
Mannitol assimilation	+	12	Naphthol-AS-BI-phosphohydrolase	-
N-acetylglucosamine assimilation	+	13	α-galactosidase	-
Maltose assimilation	+	14	β-galactosidase	+
Potassium gluconate assimilation	+	15	β-glucuronidase	-
Capric acid assimilation	-	16	α-glucosidase	+
Adipic acid assimilation	-	17	β-glucosidase	+
Malic acid assimilation	+	18	n-Acetyl-β-glucosaminidase	-
Citric acid assimilation	+	19	α-mannosidase	-
Phenylacetic acid assimilation	-	20	α-fucosidase	-

Table S3. Biosynthetic gene clusters (BGCs) predicted by AntiSMASH v 7.1 in the *Streptomyces* sp. HR58 genome

Region	Type	From	To	Most similar known cluster		Similarity
Region 1	butyrolactone	61.963	72.907	coelimycin P1	Polyketide:Modular type I polyketide	8%
Region 2	terpene	104.315	126.528	geosmin	Terpene	100%
Region 3	NRP-metallophore,NRPS	190.396	290.614	griseobactin	NRP	100%
Region 4	T3PKS	366.439	407.557	naringenin	Polyketide:Type III polyketide	100%
Region 5	LAP,thiopeptide	641.381	671.571	kanamycin	Saccharide	8%
Region 6	NRPS	699.742	743.995	collismycin A	NRP+Polyketide:Modular type I polyketide	11%
Region 7	terpene	1.062.101	1.083.177	steffimycin D	Polyketide:Type II polyketide+Saccharide:Hybrid/tailoring saccharide	19%
Region 8	ectoine	1.517.172	1.527.570	ectoine	Other	100%
Region 9	NRPS,RRE-containing,lanthipeptide-class-ii	2.164.924	2.228.666	WS9326	NRP	10%
Region 10	lanthipeptide-class-ii,lanthipeptide-class-iii	2.557.139	2.588.844			
Region 11	NI-siderophore	2.654.508	2.684.286	desferrioxamin B	Other	100%
Region 12	ectoine	3.474.206	3.484.616	ectoine	Other	75%
Region 13	NRPS,T1PKS,arylpolyene,butyrolactone,ectoine	4.238.862	4.381.337	skyllamycin D/skyllamycin E	NRP	97%
Region 14	NRPS	4.462.322	4.507.442	albomycin delta2	NRP	10%
Region 15	NRPS-like,T1PKS	5.297.232	5.352.442	WS9326	NRP	7%
Region 16	thiopeptide,LAP	5.356.064	5.384.967	bombyxamycin A/bombyxamycin B	Polyketide	11%

Region 17	melanin	5.399.526	5.409.948	melanin	Other	100%
Region 18	lanthipeptide-class-iii	5.453.850	5.476.480	AmfS	RiPP:Lanthipeptide	100%
Region 19	triceptide	5.653.776	5.675.184			
Region 20	terpene	5.865.046	5.886.053			
Region 21	NI-siderophore	6.280.772	6.313.493	kinamycin	Polyketide	19%
Region 22	hydrogen-cyanide	6.386.430	6.399.282	aborycin	RiPP	21%
Region 23	NRPS,T2PKS,oligosaccharide	6.475.410	6.554.841	warkmycin CS1/warkmycin CS2	Polyketide+Saccharide:Hybrid/tailoring saccharide	97%
Region 24	RiPP-like	6.632.020	6.643.405			
Region 25	terpene	6.855.421	6.876.731	2-methylisoborneol	Terpene	100%
Region 26	terpene	7.293.396	7.319.969	hopene	Terpene	69%
Region 27	NRPS-like,NRPS	7.350.430	7.401.553	nucleocidin	Other	47%
Region 28	T1PKS,NRPS,RiPP-like	7.441.901	7.548.089	SGR PTMs/SGR PTM Compound b/SGR PTM Compound c/SGR PTM Compound d	NRP+Polyketide	100%
Region 29	T1PKS	7.636.142	7.681.973	C-1027	Polyketide	16%
Region 30	NRPS,T1PKS	7.685.860	7.802.684	gausemycin A/gausemycin B	NRP+Saccharide	10%
Region 31	RiPP-like	7.821.405	7.832.778	streptamidine	RiPP:Other	58%
Region 32	melanin	7.833.550	7.844.050	melanin	Other	100%
Region 33	T3PKS,NRPS	7.876.814	7.983.006	cyclofaulknamycin	Polyketide	25%
Region 34	transAT-PKS,NRPS-like,lanthipeptide-class-iv	8.008.244	8.080.779	cycloheximide	Polyketide:Trans-AT type I polyketide	94%
Region 35	T1PKS,NRPS	8.133.187	8.186.074	balhimycin	NRP	8%

Table S4. Descriptive and inferential statistical analysis of fungal growth inhibition by HR58 and the positive-control strain *S. lacaronensis* OE57

Fungal isolate	n	Control mean	Control SD	HR58 mean	HR58 SD	HR58 inhibition (%)	HR58 Welch p	HR58 Holm p	HR58 sig.	OE57 mean	OE57 SD	OE57 inhibition (%)	OE57 Welch p	OE57 Holm p
A. alternata	3	3,4583	0,1774	1,3667	0,1443	60,4819	0,0001	0,0019	Yes	0,4125	0,0125	88,0723	0,0011	0,0064
C. luteo-olivacea	3	1,4000	0,0000	0,6333	0,0577	54,7619	0,0019	0,0094	Yes	0,7750	0,0866	44,6429	0,0063	0,0134
D. macrodydima	3	3,0833	0,0289	1,7000	0,0000	44,8649	0,0001	0,0019	Yes	0,8917	0,0764	71,0811	0,0001	0,0016
D. seriata	3	2,9750	0,0661	1,8917	0,0520	36,4146	0,0000	0,0008	Yes	1,3000	0,1146	56,3025	0,0001	0,0019
F. fujikuroi	3	3,7333	0,0946	2,1750	0,2385	41,7411	0,0033	0,0131	Yes	0,6933	0,0404	81,4286	0,0000	0,0009
F. oxysporum	3	3,8417	0,1809	1,9625	0,0992	48,9154	0,0005	0,0046	Yes	0,5417	0,0629	85,9002	0,0003	0,0034
P. chlamydospora	3	2,2917	0,0289	1,2250	0,2000	46,5455	0,0103	0,0134	Yes	1,0417	0,0629	54,5455	0,0001	0,0019
P. richardsiae	3	2,3333	0,0520	0,8917	0,1010	61,7857	0,0002	0,0025	Yes	0,5417	0,0144	76,7857	0,0001	0,0019
R. solani	3	4,2000	0,0000	1,3417	0,0144	68,0556	0,0000	0,0002	Yes	1,0083	0,1283	75,9921	0,0005	0,0048
T. olida	3	2,5017	0,0448	1,8583	0,0520	25,7162	0,0001	0,0019	Yes	1,1833	0,1843	52,6982	0,0045	0,0134
V. dahliae D0913	3	3,4375	0,0250	0,7500	0,0820	78,1818	0,0001	0,0019	Yes	0,1208	0,0289	96,4848	0,0000	0,0000
V. dahliae ND1113	3	3,7958	0,1697	0,7250	0,0250	80,9001	0,0008	0,0058	Yes	0,1792	0,0072	95,2799	0,0007	0,0058
V. dahliae V937I	3	2,8667	0,0289	0,9833	0,0289	65,6977	0,0000	0,0000	Yes	0,0667	0,0764	97,6744	0,0000	0,0009

Table S5. Descriptive and inferential statistical analysis of inhibition-zone diameters against *Verticillium dahliae* in the HR58-derived AgNP dose-response assay

Analysis	Statistic	Raw p	Comparison	Holm-adjusted p	Significant	Method
Global dose effect	19,3691149	0,00358361	Water + six AgNP doses		Yes	Kruskal–Wallis
All conditions	22,0293125	0,00251102	Including AgNO3		Yes	Kruskal–Wallis
vs water		1	0.5 µg	1	No	Dunn–Holm
vs water		1	1 µg	1	No	Dunn–Holm
vs water		0,2717157	20 µg	0,8151472	No	Dunn–Holm
vs water		0,0433829	40 µg	0,1735318	No	Dunn–Holm
vs water		0,0106276	100 µg	0,0531379	No	Dunn–Holm
vs water		0,00148021	190 µg	0,00888124	Yes	Dunn–Holm
vs AgNO3		0,4576944	20 µg	1	No	Dunn–Holm
vs AgNO3		0,8585379	40 µg	1	No	Dunn–Holm
vs AgNO3		0,4758812	100 µg	1	No	Dunn–Holm
vs AgNO3		0,1812985	190 µg	0,7251941	No	Dunn–Holm

Table S6. Putative genomic traits of *Streptomyces* sp. HR58 potentially associated with plant growth promotion, antifungal activity and extracellular AgNP formation.

Functional category	Trait/process	Bakta/antiSMASH annotation	HR58	ATCC 11523
Plant-associated traits	ACC deaminase	1-aminocyclopropane-1-carboxylate deaminase	2	2
Plant-associated traits	Organic-phosphorus mineralization	Phosphodiesterase/alkaline phosphatase D (PhoD)	3	2
Plant-associated traits	Phosphate metabolism	Broad-specificity phosphatase PhoE	8	8
Plant-associated traits	Secreted phosphatase activity	Secreted phosphatase, PhoX family	3	3
Iron acquisition / PGPR-related	Siderophore biosynthesis	Siderophore synthetase component IucA/IucC/SbnC	1	1
Iron acquisition / PGPR-related	Desferrioxamine-like biosynthetic region	Desferrioxamine B-like siderophore BGC (80% similarity to the known cluster)	1	1
Antifungal potential	Chitin degradation	Chitinases, including GH18/GH19 and exochitinase annotations	9	8
Antifungal potential	β-glucan degradation	β-glucanase, GH16 family	3	3
Antifungal potential	Cellulose/cellobiose degradation	Cellulase/cellobiase CelA1	2	2
Antifungal potential	Extracellular proteolysis	Serine proteases, subtilisin family	7	7
Potential contribution to AgNP formation	Flavin-dependent redox activity	NAD(P)H-dependent flavin oxidoreductase YrpB family	4	4
Potential contribution to AgNP formation	Iron-associated reductase activity	NADPH-dependent ferric-siderophore reductases	7	6
Potential contribution to AgNP formation	Nitroreductase/deazaflavin redox systems	Nitroreductase and nitroreductase-domain proteins	15	10
Potential contribution to AgNP formation	Thiol-dependent redox homeostasis	Thioredoxin and thioredoxin-related proteins	11	14
